# Supplementary material for: A Critical View of the Application of the APEX Software (Aqueous Photochemistry of Environmentally-Occurring Xenobiotics) to Predict Photoreaction Kinetics in Surface Freshwaters
Source: Molecules. 2019 Dec 18;25(1):9. doi: 10.3390/molecules25010009 (PMC7017383; doi:10.3390/molecules25010009)
Supplement: Supplementary file 1 [file molecules-25-00009-s001.zip › 3.2.4_gcc-4.4.0.zip!/3.2.4_gcc-4.4.0/3.2.4_gcc-4.4.0/share/octave/packages/symbolic-1.0.9/doc/symbolic.html]

Octave Symbolic Manipulation Toolbox

The Octave Symbolic Manipulation Toolbox is based upon  GiNaC
.   The goal is to simply provide the capabilities of GiNaC in
the easy to use environment provided by Octave.

Limitations/Features
  
Currently there is no support for symbolic matrices. I think it would
require a few changes to the parser to do it nicely: for example: 
sym\_matrix = [x+1, x+5; x^2+4,x^2+2\*x+1];  I could make a function
like sym\_matrix(the\_rows,the\_columns,x+1, ... )  that returned a symbolic
matrix but this would be a bit of a kludge.

In order to do exact arithmetic you need to deal with strings and the
vpa command.  For example:  vpa("1")/vpa("7") is represented
internally as exactly 1/7.   However,  vpa("1")/7 or 1/vpa("7") 
is an approximation to 1/7 that is accurate to roughly the accuracy of
the current value of digits.

GiNaC throws exceptions when there are problems with computations.  
I handle some of them at this time, but I do not handle all of them.  
This can cause octave to terminate prematurely.   For example,
try vpa("1")/vpa("0").   This will eventually be fixed.

Download and install

You will need to install cln-1.0.1, GiNaC-0.8.0 and octave-2.1.33 or
later to use this package.  You may be able to get by with an earlier
version of octave if you compiled without the "-fno-rtti -fno-exceptions"
options.   This package uses both exceptions and run-time type
identification.    There is an INSTALL file in the package
which will tell you how to install the package.

Functions
  
Below I provide a list of function that I have implemented or have
plans to implement as of the latest release .  If the function name
is green then
the function is implemented and to the best of my knowledge there are no
problems with it.  If the function is red
then it has not been implemented yet. 
If the function is blue
then it has been implemented but is known not to work correctly. 
Blues will appear only very rarely.

- vpa  - create a variable precision arithmetic
  variable from a string, double, or an appropriate expression.
- sym  - create a symbolic variable
- is\_vpa  - returns true if an object is
  a vpa object
- is\_sym  - return true if the argument
  is a symbolic variable
- is\_ex  - returns true if an object a
  symbolic expression (i.e.  x+y)
- to\_double  - convert a vpa, ex or string
  to a double value.
- to\_char  - convert a vpa, ex to a string.
- digits  - set or view the number of digits
  that newly created vpa object should have
- Abs  - Absolute value
- csgn  -
- Sqrt  - Sqrt(x)  => x^(vpa(1)/2)
  or x^(1/vpa(2))
- Cos  - the cosine of a sym, vpa , or
  ex variable
- Sin - the sine of a sym, vpa , or ex variable
- Tan - the tangent of a sym, vpa , or ex variable
- aCos  - the inverse cosine of a sym,
  vpa , or ex variable
- aSin  - the inverse sin of a sym, vpa
  , or ex variable
- aTan  - the inverse tangent of a sym,
  vpa , or ex variable
- aTan2  -
- Cosh  - the hyperbolic cosine of a sym,
  vpa , or ex variable
- Sinh  - the hyperbolic sine of a sym,
  vpa , or ex variable
- Tanh  - the hyperbolic tangent of a sym,
  vpa , or ex variable
- aCosh - the inverse hyperbolic cosine of a
  sym, vpa , or ex variable
- aSinh - the inverse hyperbolic sine of a sym,
  vpa , or ex variable
- aTanh  - the inverse hyperbolic tangent
  of a sym, vpa , or ex variable
- Exp  - the cosine of a sym, vpa , or
  ex variable
- Log  - the cosine of a sym, vpa , or
  ex variable
- Zeta  -
- Tgamma  -
- Lgamma  -
- Beta  -
- Factorial  -
- Binomial  -
- Order -
- subs - perform a substitution in an expression
- differentiate  - differentiate an expression
- expand  -  multiply all of the terms
  in an expression out:  (x+y)\*(x+z) => x^2+x\*y+x\*z+y\*z
- collect  - collect similar terms in an
  already expanded expression
- coeff  - return the nth coefficient in
  a polynomial
- lcoeff  - leading coefficient of a polynomial
  (4x^2+2x+5 => 4)
- tcoeff  - trailing coefficient of a polynomial
  (4x^2+2x+5 => 5)
- degree  - The degree of a polynomial
  (i.e. x^2+2x+1 => 2)
- ldegree  - The low degree of a polynomial
  (i.e. x^2+2x+1  => 0)
- quotient  -
- remainder  -
- premainder  -
- unit  -
- content  -
- primpart  -
- Gcd  - greatest common denominator of
  a polynomial expression
- Lcm  - least common multiple of a polynomial
  expression
- numer  -
- denom  -
- normal  - ?
- to\_rational  -
- Series  -
- Pi  - pi evaluated to the current value
  of digits accuracy.
- splot  -plot a symbolic functin over
  a range of values
- Saving of expressions, Retrieving of expressions
